# Ethical issues in autologous stem cell transplantation (ASCT) in advanced breast cancer: A systematic literature review

**DOI:** 10.1186/1472-6939-12-6

**Published:** 2011-04-15

**Authors:** Sigrid Droste, Annegret Herrmann-Frank, Fueloep Scheibler, Tanja Krones

**Affiliations:** 1 Institute for Quality and Efficiency in Health Care (IQWiG), Cologne, Germany; 2 Institute of Biomedical Ethics, University Hospital, Zurich, Switzerland

## Abstract

**Background:**

An effectiveness assessment on ASCT in locally advanced and metastatic breast cancer identified serious ethical issues associated with this intervention. Our objective was to systematically review these aspects by means of a literature analysis.

**Methods:**

We chose the reflexive Socratic approach as the review method using Hofmann's question list, conducted a comprehensive literature search in biomedical, psychological and ethics bibliographic databases and screened the resulting hits in a 2-step selection process. Relevant arguments were assembled from the included articles, and were assessed and assigned to the question list. Hofmann's questions were addressed by synthesizing these arguments.

**Results:**

Of the identified 879 documents 102 included arguments related to one or more questions from Hofmann's question list. The most important ethical issues were the implementation of ASCT in clinical practice on the basis of phase-II trials in the 1990s and the publication of falsified data in the first randomized controlled trials (Bezwoda fraud), which caused significant negative effects on recruiting patients for further clinical trials and the doctor-patient relationship. Recent meta-analyses report a marginal effect in prolonging disease-free survival, accompanied by severe harms, including death. ASCT in breast cancer remains a stigmatized technology. Reported health-related-quality-of-life data are often at high risk of bias in favor of the survivors. Furthermore little attention has been paid to those patients who were dying.

**Conclusions:**

The questions were addressed in different degrees of completeness. All arguments were assignable to the questions. The central ethical dimensions of ASCT could be discussed by reviewing the published literature.

## Background

A systematic review on the effectiveness of autologous stem cell transplantation (ASCT) in locally advanced and metastatic breast cancer by the German Institute for Quality and Efficiency in Health Care (IQWiG) [1] indicated important ethical issues associated with the use of this technology in clinical practice.

Among others, ASCT is a potentially curative treatment option in locally advanced and metastatic breast cancer, a life-threatening disease. Immediately after high-dose chemotherapy, a woman is transfused with their own stem cells, which were harvested before high-dose chemotherapy begun. Apart from damaging tumor cells, high-dose chemotherapy also damages vital hematopoietic stem cells, so ASCT replaces the destroyed bone marrow. The potential benefit of extending patient overall survival or disease-free survival is accompanied by severe toxicities such as infections, bleeding, secondary malignancies, infertility etc. - including the risk of therapy-induced death.

### Objectives

In light of the existing benefit-harm balance, we decided to systematically review the ethical issues related to the use of this technology by analyzing the published literature. Because in ethics systematic reviews are not as widespread as in medicine, our aim was to reveal the feasibility of conducting a systematic review in ethics. This was done by analyzing the relevant literature.

## Methods

We undertook a systematic review of the published literature on ethical issues related to ASCT in locally advanced and metastatic breast cancer patients. Our workflow consisted of the following working steps:

-Choosing the methodological approach to review ethical issues related to health technologies,

-Searching for the existing publications by doing a systematic literature search,

-Selecting the retrieved documents by applying predefined inclusion and exclusion criteria,

-Abstracting the relevant data (arguments) and assessing their validity,

-Synthesizing the qualitative data,

-Addressing Hofmann's questions.

The review process was done by a multidisciplinary team: one author conducted the information retrieval (incl. pre-screening) while screening, reviewing and summarizing the results were done by all authors - inter alia an ethicist.

### Methodological approach

Several methods do exist to consider ethical issues in HTA [2]. We chose the reflexive Socratic approach for our analysis by using Hofmann's question list as a pilot scheme [3]. This question list includes 33 questions on ethical issues and values of health technologies, their use and their assessment. In detail, these are:

-Moral issues (harm to patients, patient autonomy, religious, social or cultural convictions),

-Questions with respect to stakeholders (such as interests of producers or users),

-Questions related to technology (such as the moral relevance of a possible existing symbolic value of a technology),

-Moral aspects of methodological choices (such as representativeness of clinical study populations for users in clinical practice),

-Questions related to technology assessment (such as moral consequences of the HTA).

Applying Hofmann's question list to ASCT resulted in a presentation of a wide variety of qualitative arguments. To present the results in a more transparent and reproducible way, we refrained from summarizing the results according to the dimensions of the question list, but rather addressed each question in detail.

### Information retrieval

We conducted a systematic, comprehensive literature search in the cited biomedical, psychological and ethics databases (January 2008, see Table 1). This process, adapted to the specific needs of information retrieval on ethical issues of health technologies, comprises systematic retrieval in addition to narrative searching such as snowballing and citation tracking [4]. The search command and strategy building facilities - particularly in the ethics databases - are restricted. Thus the retrieval partly included a pre-screening of the bibliographic data by means of defined inclusion and exclusion criteria.

**Table 1:** List of bibliographic databases which were included in the information retrieval process

Biomedicine	**International**: Cochrane Database of Methodology Studies, Cochrane Central Register of Controlled Trials, Cochrane Database of Systematic Reviews, BIOSIS, EMBASE, MEDLINE, NHS DARE, NHS HTA, SCISEARCH **National**: CCMED
**Nursing**	**International**: CINAHL

**Psychology**	**International**: PSYCINFO **National**: PSYNDEX

**Social Sciences**	**International**: SOCIAL SCISEARCH **National**: SOFIS

**Health Economics**	**International**: NHS EED

**Ethics**	**International**: EURETH, ETHX **National**: BELIT

**Monographs**	**International**: LocatorPlus **National**: Karlsruher virtueller Katalog (KVK)

**Publisher databases**	Elsevier (ScienceDirect), Thomson Reuters (Journals@OVID), Karger, Kluwer, Springer, Thieme

These pre-screening criteria were based on background knowledge of the technology. The searches were very sensitive in order to avoid excluding any possibly relevant publication. No language restrictions were introduced. The information retrieval process has been published previously in detail [5].

#### Pre-screening inclusion criteria (relevant to Ethics databases)

-Publications on stem cell research or use,

-Publications of the German National Ethics Council, key publications of the European Union on stem cell research and therapy or substantial work on pros and cons of stem cell research,

-Publications on adverse events/harms of stem cell therapy,

-Publications on stem cell therapy regardless of kind and origin of cells (embryonic, autologous, cord blood etc.),

-Health economic publications on stem cell transplantation, -Publications on survival or quality of life post-stem cell transplantation,

-Substantial monographs suggesting matters relevant to stem cell therapy.

#### Pre-screening exclusion criteria

-Journal articles in which the title solely refers to embryonic stem cell research, importing of embryonic stem cells, children, or indications other than breast cancer,

-Publications recognizable as basic research (e.g. dealing with chimerism),

-Publications on the position of religious groups regarding stem cell research,

-Publications exclusively linked to regions outside of Europe,

-Newspaper articles or other non-scientific journals.

### Selecting the retrieved information

Two reviewers selected the identified documents based on title and abstract (where available). In the case of dissent, a third reviewer was consulted. If relevant, or in the case of doubt, full-text articles were obtained. Included were all documents that fulfilled one or more inclusion criteria or did not fulfill one or more exclusion criteria. Inclusion and exclusion criteria for selection were as follows:

#### First and second screening inclusion criteria

-Publications on stem cell research,

-Publications by the German National Ethics Council, key publications of the European Union on stem cell research and therapy - or substantial work on pros and cons on stem cell research,

-Publications on stem cell therapy (inclusive of adverse events/harms),

-Health economic publications on stem cell transplantation, balancing benefit and harm,

-Publications on quality-of-life in patients after ASCT.

#### First and second screening exclusion criteria

-Publications which did not explicitly deal with ethical issues of ASCT in breast cancer

-Daily newspaper articles or other non-scientific journals,

-Publications on embryonic stem cell therapy containing no arguments on ethical issues related to stem cell transplantation in breast cancer patients,

-Publications on stem cell therapy in children or indications other than breast cancer with no relevance to ethical issues in breast cancer patients,

-Publications on results of basic research

-Publications on descriptive pre-post health quality-of-life studies without arguments related to ethical issues

-Publications on the position of religious groups regarding stem cell research containing no information on the position of these groups regarding autologous stem cell therapy,

-Publications exclusively linked to regions outside of Europe, for example stem cell therapy in the Philippines

The included full-text articles were then analyzed with regard to qualitative arguments on ethical issues. The relevant arguments were assembled and extracted by using excerpts or paraphrasing and assessed for their validity and relevance. The synthesized arguments were then assigned to the dimensions of Hofmann's question list and the related question/s [3].

Finally, Hofmann's 33 questions were addressed by using these arguments. Only a small number of questions, such as on the interests of the reviewers (Q30), could not be addressed by literature analysis. These questions were answered by the reviewers themselves.

## Results

**Figure 1:**
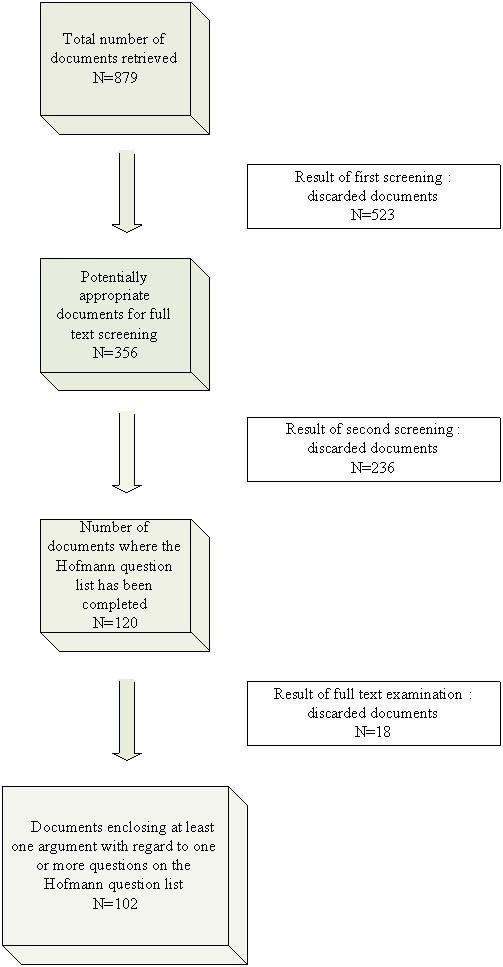
**Flowchart on the selection process of the information retrieval result**.

Searching the 27 databases we identified 879 titles, 356 of which were screened by using title and abstract. Of these 120 were screened in full text and considered relevant to our research question. Finally, after adjusting for remaining duplicates (e.g. duplicate publications in different languages) and for those documents later found to meet the exclusion criteria, the remaining 102 publications comprised one or more arguments with regard to one or more questions in the checklist (Figure 1). We used Hofmann's question list to analyze these documents.

Publication language was mainly English (88 publications = 86%), followed by German (11 publications = 11%), French (2 publications = 2%) and Swedish (1 publication = 1%). Some publications comprised only a few sentences on ethical issues. The main objectives of the articles, and fields of the authors, were wide-ranging (see Table 2).

**Table 2:** Main objectives of the included 102 articles and specialism of the authors

Main objective/specialism	**Number of publications**^**1**^
Ethical issues	35 (34%)
Ethics + Law/Quality of life/Psychology/Health Economics/Health Policy	4 (4%)
Legal issues	15 (15%)
Law + Health Policy	2 (2%)
Medical issues/effectiveness evaluation	12 (12%)
Quality of life research	10 (10%)
Quality of life + psychology	3 (3%)
Psychological issues	9 (9%)
Health Economics	4 (4%)
Health Policy	4 (4%)
Socio-cultural issues	1 (1%)
Others	3 (3%)

^1 ^Some publications referred to more than one single main objective.

Study design also varied among the 102 included publications (see Table 3). Levels of evidence were not assigned to the literature.

**Table 3:** Publication design of the included 102 articles

Publication Design	Number of publications
**Review literature**	
HTA report	4 (4%)
Systematic review	6 (6%)
Review article	26 (25%)
Expert statement/Newspaper article	20 (20%)
Opinion (parliamentary/scientific society etc.)	5 (5%)
Dissertation	2 (2%)
Editorial	5 (5%)
Commentary/letter	8 (8%)
	
**Primary literature**	
Randomized controlled trial	2 (2%)
Prospective observational/cohort study	4 (4%)
Retrospective observational/cohort study	7 (7%)
Prospective longitudinal study	4 (4%)
Cross-sectional study	2 (2%)
Qualitative research study	5 (5%)
Case report	2 (2%)

Overall, 30 of the 33 questions were addressed by the identified publications: Question 8 (Q8) and question 32 (Q32) were not addressed, while question 30 (Q30) had to be answered by the authors of this review. For detailed information see figure 1.

### Hofmann's Question list

We found some relevant arguments in the published literature for all Hofmann's dimensions, each of which was concerned with the use of ASCT in breast cancer patients. These dimensions included "moral issues" (questions1-16), "questions with respect to stakeholders" (questions 17-20), "questions related to technology" (questions 21-23), "moral aspects of methodological choices" (questions 24-28) and "questions related to technology assessment" (questions 29-33). In addition to patients, physicians, nurses and relatives, societal values were affected by the use of this technology. According to the different weight of the dimensions and questions, the frequency of addressing them by publications varied widely (Table 4).

**Table 4:** Number of publications with arguments related to the dimensions and questions of Hofmann's question list

Dimension/Question	Number of publications with related arguments (N = 102)
**Moral issues**	**81**

Q1 What are the morally relevant consequences of the implementation of the technology?	18

Q2 Does the implementation or use of the technology challenge patient autonomy?	24

Q3 Does the technology violate or interfere with basic human rights in any way?	2

Q4 Does the technology challenge human integrity?	9

Q5 Does the technology challenge human dignity?	4

Q6 Will there be a moral obligation related to the implementation and use of a technology?	11

Q7 Does the technology challenge social values and arrangements?	3

Q8 Does the widespread use of the technology change our conception of certain persons?	0

Q9 Does the technology contest religious, social or cultural convictions?	1

Q10 Can the use of the technology in any way challenge relevant law?	2

Q11 How does the assessed technology relate to more general critiques of modern medicine?	16

Q12 Are there any related technologies that have turned out to be morally challenging?	2

Q13 Does the technology in any way challenge or change the relationship between physician and patient?	5

Q14 How does the implementation of the technology affect the distribution of health care?	23

Q15 How does the technology contribute to or challenge professional autonomy?	2

Q16 Can the technology harm the patient?	23

**Questions with respect to stakeholders**	**19**

Q17 What patient group is the beneficiary of the technology?	10

Q18 Are there third party agents involved?	5

Q19 What are the interests of the users of the technology?	7

Q20 What are the interests of the producers of technology (industry, universities)?	1

**Questions Related to Technology**	**11**

Q21 Are there moral challenges related to components of a technology that are relevant to the technology as such?	1

Q22 What is the characteristic of the technology to be assessed?	2

Q23 Is the symbolic value of the technology of any moral relevance?	9

**Moral Aspects of Methodological Choices**	**35**

Q24 Are there morally relevant issues related to the choice of end points in the assessment?	19

Q25 Are there morally relevant issues related to the selection of meta-analysis and studies to be included in the HTA?	9

Q26 Are the users of the technology in the studies representative of the users that will apply it in clinical practice?	5

Q27 Are there morally relevant aspects with respect to the level of generalisation?	2

Q28 Are there moral issues in research ethics that are important to HTAs?	18

**Questions Related to Technology Assessment**	**9**

Q29 What are the reasons that this technology is selected to be assessed?	2

Q30 What are the interests of the persons participating in the technology assessment?	0

Q31 At what time in the development of the technology is it assessed?	1

Q32 Are there related technologies that have or have not been assessed?	4

Q33 What are the moral consequences of the HTA?	2

(N = 102)

We synthesized the relevant arguments from the published literature addressing each question of Hofmann's question list separately. The answers to Q12, Q29 to Q33 are partly or completely written by the authors of this review. All other parts are summaries and paraphrases of the published literature.

#### Questions "Moral issues" (Q1 to Q16)

##### Q1 What are the morally relevant consequences of the implementation of the technology?

Several ethical issues have been addressed in numerous articles with regard to autologous stem cell transplantation in breast cancer patients [6-24]. In the late 1980s, ASCT appeared to be a highly promising treatment for high risk and metastatic breast cancer patients and was introduced into clinical practice without further evaluation in clinical trials. Belanger et al. [25] described in a 1991 survey that 80% of oncologists claimed that they would offer ASCT to their patients.

However, since the new procedure also caused toxic side effects, patients were faced with the decision to consent to a hopefully life-prolonging but also potentially fatal treatment. This decision had to be taken in the context of extensive promotional media campaigns and without reliable data or independent patient information. In addition, in the late 1990s, the first randomized trials were started. However, due to early introduction of ASCT into clinical practice only a small number of patients received the treatment within clinical trials. The majority of the first investigational trials did not showing any benefit of ASCT in terms of overall survival. Moreover, the only two trials which showed benefit in survival were found to be fraudulent (Bezwoda fraud). Premature introduction into clinical practice, overly optimistic presentation of preliminary results in the media and the subsequent fraudulent trial had significant effects on further research and the doctor-patient relationship. Recruiting patients for further clinical trials became even more difficult due to lack of trust in oncology and the trial investigators. Sledge et al [22] pointed to the possibility of potential benefit of ASCT in spite of the falsified data. Recent meta-analyses confirmed this assumption by showing a marginal effect in prolonging disease-free survival [1,26-28]. Nevertheless, ASCT in breast cancer remains a stigmatized technology.

##### Q2 Does the implementation or use of the technology challenge patient autonomy?

ASCT may change patient autonomy in both directions [7,8,13,23,29-49]. While referred to as the ultimate therapeutic option if all other alternatives have failed (extended autonomy), it is associated with severe side effects and a high risk of mortality (reduced autonomy). Many of the identified papers stressed the need for detailed information on benefit and risks of ASCT before consent is obtained [40,46]. Some authors criticize that patients are insufficiently informed and patient understanding is not ensured [30]. Patient autonomy can only be improved if decision-making results from substantial, reliable and comprehensible patient information. Moreover, many of the patients experienced substantial economic constraints, since many parts of the therapy are not reimbursed by their health plan [43]. Further issues of patient autonomy and informed consent are discussed in Q3, Q4, Q11, Q16 and Q23.

##### Q3 Does the technology violate or interfere with basic human rights in any way?

Some issues relating to human rights were found in the literature on the application of ASCT itself and its history and implementation process. Examples of the latter:

-violation of informed consent before treatment,

-specific vulnerability of the population undergoing treatment towards serious toxicities,

-using ASCT when more effective and safer procedures exist, and

-(of minor concern in ASCT) funding of treatment with or research on human stem cells 

are all in violation of basic human rights such as autonomy and treating palliative patients with dignity [50]. Furthermore, beneficial therapy in a life-threatening situation might be impeded, since, to date, it is unknown whether there is any evidence of a benefit of ASCT for some subgroups of breast cancer patients [23].

##### Q4 Does the technology challenge human integrity?

By using human cells for ex vivo manipulation and therapeutic purposes, stem cell transplantation has changed the image of humans and interfered with public values [6,51,52]. The commercialization of stem cell transplants or patenting of isolating adult stem cells (stem cells modified between the time of harvesting and use - thus warranting a change of property rights and, enabling them to be used by third parties) also challenges human integrity [32,53]. Moreover, in palliative situations some patients undergo ASCT to avoid anything that could be interpreted as a rebellion against their caregivers. Being highly dependent on them, these patients may consent to high-risk therapies that might do more harm than good [54]. Whether ASCT challenges the individual's human integrity depends on the individual's values and the extent of informed consent. Presenting phase II results and then falsified data prevented the patients from preserving their integrity [39,47,49].

##### Q5 Does the technology challenge human dignity?

This question also depends on the fundamental cultural interpretations of the concept of human dignity and its representation in constitutional law [55]. This includes a reflection on whether commercialization and patenting of human stem cells or their isolation procedure or research on human stem cells (a minor problem in autologous transplantation) is compatible with a universal or specific concept of human dignity [32,53,56]. Results of stem cell research and their presentation by media encouraged the public's belief that curative treatments for chronic diseases in general would soon be available [55]. Moreover, the autologous variant of stem cell transplantation carries some risk of challenging human dignity, e.g. it has raised hope for an existing treatment option and ultimately a cure, thus preventing a dignified death [56].

##### Q6 Will there be a moral obligation related to the implementation and use of a technology?

There has been an important moral obligation attached to ASCT regarding the debate on the "imperative of technology" in terms of imperative of possibilities [57]: the new high technology (thought to be a true innovation) with the potential to benefit women with a life-threatening disease raised new hope for patients and physicians [18,58,59]. The basic problem was caused by the premature implementation before evaluation and clinical practice use in the 1990s. Implementation was based on phase II evidence [18,34]. In ASCT, it was not evidence but enthusiasm, hope, emotions and finally court decisions that opted for implementation before evaluation [11,24,50,60]. This implementation on limited evidence impeded the identification of those patient groups that would benefit (most) and the research on alternatives [61,62].

##### Q7 Does the technology challenge social values and arrangements?

There are few references to the challenge of social values by ASCT. When a "last chance therapy" emerges, the hitherto fatal disease seems to become manageable and near-possible death becomes less proximate or is still considered avoidable by patients and the public. As a consequence, patients postpone preparing for death [34,42].

Another point of interest is the financing of experimental therapies such as ASCT based on health insurance funds and their specific criteria. Diseases receive a different level of significance if treatment options are not covered by insurers in general, but could be covered by them were fulfilling certain conditions to be meet, such as like qualified documentation, enrolment in controlled therapeutic studies, etc. as in the case of ASCT [63]. See also Q18.

##### Q8 Does the widespread use of the technology change our conception of certain persons?

No indication could be found in the literature on changes in the conception of women with breast cancer with regard to ASCT. However, there may be some changes, which were not discussed by literature.

##### Q9 Does the technology contest religious, social or cultural convictions?

No indication could be identified on convictions of any religious, social or cultural group with regard to ASCT. Moreover, Jehovah's Witnesses did not offer any objection to this autologous variant of stem cell transplantation [64]. There may be some religious, social or cultural groups outside of the German/European focus with relevant convictions regarding ASCT which were undiscovered by the information retrieval and the defined inclusion and exclusion criteria.

##### Q10 Can the use of the technology in any way challenge relevant law?

Autologous stem cell transplantation did not change the relevant law - contrary to embryonic stem cell research. But the judiciary played a decisive role in ASCT history - particularly in the USA. The legal and political pressure led most health insurance funds to pay for ASCT by the mid-1990s [18]. As most of the later trials did not show distinct superiority of ASCT compared to conventional chemotherapy in breast cancer, coverage policy by insurers become more restrictive, i.e. denying coverage [24].

##### Q11 How does the assessed technology relate to more general critiques of modern medicine?

One of the main problems of ASCT discussed in the literature is the conflict of values between rapid access to an experimental therapy for those patients for whom conventional therapies offer little hope, and attempts to protect patients from therapies that are unproven (or expensive or potentially harmful) or even inferior to existing therapies [58,65]. This is a general conflict between technical and medical progress, carrying the danger of implementing an experimental therapy as standard therapy [19,65-67]. It includes an inherent conflict between the obligation of a physician to treat optimally and the publics need to have a procedure's effectiveness evaluated and protect the integrity of the evaluation process [41,58,68].

Furthermore, according to Davies [69], the media and general public did not seem to wish to recognize the limitations of medicine and the inability of physicians to cure every disease. Therapeutic concepts based on human stem cells contribute to an image of "repairing medicine" to injuries with disease character [70]. Furthermore, modern medicine pursues the "tendency to focus on the status of the body rather than the patient as a holistic person".

Hence, research funding is more focused on improving disease outcome than it is on the demands of patients who are dying [71] and on the tolerance and preferences of those individuals [51], thus drawing attention away from more appropriate efforts to minimize symptoms and enhance the quality of life for terminally ill patients and their families [35].

If experimental treatment is available outside of trials it is difficult to recruit patients for clinical trials. In ASCT trials the recruiting phases had to be greatly extended. As a result, it became necessary to legitimize evaluation, while the evaluation process progressed more slowly than the diffusion of clinical use. In addition no subgroup analysis has been done [19,23,35,68]. Lambird [41] argues that false hopes are raised in patients offering to participate in research protocols because the enthusiasm of the investigators often obstructs precise clinical research. In ASCT, conclusions have been drawn before evaluating phase III data [37]. In contrast, Rushing [72] poses the question: is it ethical to recruit patients for phase III trials to confirm the benefits of survival that had previously been demonstrated in phase II trials?

Finally, important lessons from ASCT related to modern medicine are the collateral effects of both implementing a technology without evaluating its effectiveness and the Bezwoda fraud. The physician-patient relationship (physicians' misguided and/or enthusiastic recommendations), patients' informed consent on the basis of manipulated data, and the resulting patient harm are of concern. This Bezwoda fraud not only contradicts the ethical principle of "first do no harm" (*primum non nocere*), it also has consequences for all oncologists and the discipline itself. It is not yet clarified whether ASCT in breast cancer might save or extend lives. Oncology has become untrustworthy and all oncologists are confronted with the falsified data. Patients become less recruitable for clinical trials. Both the University of Johannesburg and South African scientists in general are affected by the association with Bezwoda. Science itself is also affected because science is based on confidence and if a scientist presents his/her data publicly, the data are accepted as fact. Unethical and dishonest clinical experiments tend to have devastating, long-term consequences on honest, ethical experiments [22].

Evans [58] is critical of an institutional deficit in the relationship between clinical researchers, attending physicians, insurers and institutions like the National Cancer Institute, and of the supervision of the evaluation of medical procedures as they move from small hypotheses to large RCTs.

##### Q12 Are there any related technologies that have turned out to be morally challenging?

Today, the most morally challenging related technology is the so-called cord blood transplantation [32]. For this type of transplantation, many issues concerning children's and parents' rights and the large field of commercialization are currently being discussed. Controversy also abounds around allogeneic stem cell transplantation [73], which exerts an even higher toxic profile than ASCT [61].

##### Q13 Does the technology in any way challenge or change the relationship between physician and patient?

Two main issues are discussed in the relevant literature [15,22,31,72,74]. After the Bezwoda fraud was published, it became almost impossible to recruit patients for further phase III trials [72] because of patients' loss of trust in their physicians and their recommendations. Moreover, the demand for independent, true and honest information has increased. Doctors might be too optimistic about the benefits of ASCT and afraid to explain the true risks of this treatment. Patients might be unrealistically optimistic [15]. Life-threatening situations and high-risk therapies are often associated with an emotional crisis in patients. This could interfere with clear communication of benefits and risks [74]. Cohen adds that isolation creates a special dependency of the patient on nurses and physicians [31].

##### Q14 How does the implementation of the technology affect the distribution of health care?

Actually, there are no distributional issues of health care. The frequency of use of ASCT in breast cancer patients now is low, but it is still an expensive treatment compared to alternatives. In the 1990s, there were strong concerns relating to coverage of treatment costs, allocation of resources, equity and justice - especially in the United States. Authors described considerable regional and local differences in the coverage of ASCT. Some insurers offered no coverage while others covered the treatment only within studies, or both within and outside of studies. Equality was not guaranteed between breast cancer patients. Due to pressure from politics, media, the public and from court decisions from the mid-1990s, almost all insurers covered ASCT - though they designated it as investigational or experimental. Thus, equality between breast cancer patients has been guaranteed, but prioritizing ASCT tied up enormous resources of the insurers and left less financial resources for other diseases/patients [10,12,15,21,34,36,37,61,65,75-79].

Publications since the Bezwoda fraud was published discuss the problem of treatment coverage without evidence of effectiveness and the need to ensure coverage of investigational or experimental therapies in studies only [6,14,24,80-82]. The general public, rather than insurers, should decide resource allocation, not insurers [83]. Research on human stem cells calls for basic social requirements, resources for supervision and the prevention of any abuse [70]. The problem of last chance therapies still remains that are ineffective, but not to cover this "may create the impression that critically-ill patients are being abandoned in their moment of need [84]".

##### Q15 How does the technology contribute to or challenge professional autonomy?

Two papers referred to this item [15,22]. Kletzel describes the potential role conflict of being a physician treating the individual patient and being an investigator interested in enrolling a patient in his/her clinical trial [15]. Sledge argues that ASCT could be an effective intervention but following the Bezwoda fraud testing this hypothesis in a new clinical trial could hardly be realized [22]. Not only patients but also physicians trust in research, and scientific publication has been affected by Bezwoda (cf. Q11). This leads to reduced professional autonomy.

##### Q16 Can the technology harm the patient?

All publications agree that ASCT contains a considerable harm potential. Insurers, as well as researchers, are reporting that ASCT is highly toxic - including treatment-related mortality and potentially fatal infections and long-term toxicities [21,70,71,77,85-87]. Besides these severe or serious adverse events, some negative impact on quality of life in the survivors is reported, such as psycho-social problems or depression symptoms [43,71,74,88]. Long-term studies suggest that younger women are more exposed to harm concerning emotional status, anxiety, depression, sleeping disorders, sexual activity, aims of life, isolation of their children, length of rehabilitation, etc. [31,89,90]. Clinical changes in ASCT by using additional growth factors and other supportive therapies may cause fewer long-term side effects [91,92].

Benefit and harm have not been considered in a balanced way. Despite the obvious risks of ASCT, possible benefits have been given closer attention than harms and little attention has been paid to those patients who were dying from ASCT [61,71].

Besides adverse events, there are the ethical principles of beneficence and to do no harm which are relevant for describing harm. These principles do not seem to have been fulfilled - particularly in the 1990s - due to the absence of sufficient patient information, true and honest information (cf. Q2) and informed decision-making abilities unaffected by stakeholder interests [19,70,78,93]. "The physician and patient should both undertake self-examination to ensure the treatment is being carried out for the right reasons [79]".

The problem of weighing patient-centered against population-centered harms by insurers is also discussed. Avoiding population-centered harms from imprudent use of shared resources leads to "coverage only for last-chance treatments that meet a threshold of established net benefit and payment for unproven therapies only in the context of controlled clinical trials, in which a patient might receive a placebo or the standard treatment [84]".

#### Questions "Questions with respect to stakeholders" (Q17 to Q20)

##### Q17 What patient group is the beneficiary of the technology?

Recent meta-analyses [1] report a marginal effect in prolonging disease-free survival, accompanied by severe harms including death. It is unknown to date if there is a greater benefit from ASCT to any subgroup of patients and, if so, whether this group would benefit more from an existing alternative. Perhaps only those women in palliative care who value any treatment higher than doing nothing would benefit from ASCT.

Reports published since 2001 discuss the right of patients to aggressive, toxic and expensive treatment - even those that have been untested and have demonstrated insufficient evidence on effectiveness and safety - if they are potentially life-saving or life-prolonging [19,60,94].

In the 1990s, when ASCT was regarded as beneficial to all or most breast cancer patients, issues of fairness and justice were discussed in the literature: independent access, distribution and inequality due to different coverage policies. These issues refer to prioritizing of ASCT among breast cancer patients and breast cancer patients versus patients with other diseases [15,45,67,95]. Authors expressed the hope of prompt clarification around the question of which patient group would benefit most and how the costs of ASCT could be estimated [86].

##### Q18 Are there third party agents involved?

There are some third party interests: "the requirements of clinical science, patient demands, physician advice to patients, physicians' beliefs and enthusiasm, patient advocacy, litigation, economics (the cost of the procedure, insurers' resistance to pay for investigational treatment), and entrepreneurial oncology, politics, the ambivalent commitment of oncology to randomized clinical trials, and how the media reported the story [19]".

Industry is interested in positive evaluation of ASCT because it controls the whole process from cell sampling via cell concentration to returning the cells to the patient. This challenges the autonomy of the non-commercial system of assembling blood products [52].

Initially, insurers claimed that ASCT was experimental or investigative and did not cover this technology outside of clinical trials. But in the late 1990s, insurers began covering ASCT because of court decisions, political, media and public pressure (judicial bias favors individual patients, not patient populations). For the insurers, coverage without evidence was judged to be cheaper than the risk of negative court decisions.

The individual patient is interested in using ASCT because women feel they benefit from it, while the societal interest is not to damage the health insurance system by requests for new cost-intensive and potentially ineffective technologies [37,45,58].

##### Q19 What are the interests of the users of the technology?

Users are primarily physicians, who fulfill the dual role of patient caretaker and researcher or study investigator (cf. Q15). This resulted, particularly in the 1990s, in conflicts of interest between benefits for the patient, personal benefit for the physician and financial demands [18,45,96]. "...there are financial incentives for holding onto patients and for doing transplants ... Autologous peripheral stem-cell transplant units are reportedly money-makers for medical centers [79]".

A progressive alliance of research and economy, public interest and time pressure increases the risk of implementing clinical trials before evidence is available. The researchers present possible results of their projects and create excessive expectations and hope among patients with their promises of rapid therapeutic success [60,70].

One paper addressed the ethical problem of not testing autologous blood components with the goal of reducing the risk of infections to staff and other patients [97].

##### Q20 What are the interests of the producers of technology (industry, universities)?

Actually, frequency of ASCT application is low. Thus, there are fewer incentives for producers and health professionals now than there were in the 1990s. There is still some interest from device manufacturers, health providers and universities and blood banks (which store the harvested blood during high-dose chemotherapy) [73]. "The non-profit agency will only charge what the stem cell unit costs, but agencies can engage in a large variety of activities which increase these costs" [98].

#### Questions "Questions Related to Technology" (Q21 to Q23)

##### Q21 Are there moral challenges related to components of a technology that are relevant to the technology as such?

To address this question on conclusions by analogy some background knowledge and input from other technologies is needed. However, unfortunately ASCT in other conditions and could not be extracted entirely from the identified publications. Hence, in the following we address some ethical issues identified to be of importance in the treatment of oncology patients.

Ethical issues of quality-of-life measurement, the value of life or of long-term quality-of-life studies in palliative care correspond to ethical issues of ASCT in breast cancer patients (e.g. health-related quality-of-life data are at high risk of bias in favor of the survivors). All 'last chance' therapies are related to certain identical ethical issues. New technologies for patients who are facing death always create new hope. In addition, all treatments - more or less aggressive -which induce death, pain or other severe adverse events entail some similar ethical issues. Moreover, values of patients who are facing death are usually different from those of physicians, nurses or relatives: They want to die with dignity. Preparing patients for death is important in ASCT but also in many other life-threatening diseases. Some of these points are explained by Kelly [71].

##### Q22 What is the characteristic of the technology to be assessed?

The most important purpose of ASCT is prolonging the survival or at least disease-free survival of breast cancer patients after all other available treatments have failed. This relates to such human and societal values as living as long as possible, free from debilitating symptoms, and in dignity. These values are partly discussed in the quality-of-life literature, but rarely in the publications on ethical issues of ASCT.

Uncertainty is the most important factor at all stages of transplantation. The purpose of preparing for ASCT treatment should be to identify all patients whose quality of life is so restricted that further treatment with ASCT could not be seen as ethically problematic.

A major conflict of ASCT is the question of how much information should be given before treatment. "Nursing and medical staff routinely failed to inform dying patients of their deteriorating condition." ... "Such denial of emotion, when not appropriately reflected upon as a necessary strategy for dealing with very uncomfortable events, avoids a context in which understanding and ways of coping may be developed [71]". Furthermore, patient characteristics are related to technology. The "patient's progress is composed of constant crises ... The patient is not either hopelessly terminal or probably curable, but rather in a 'high stakes' limbo and vacillations in hope, fears and anger emerge throughout the process [44]".

##### Q23 Is the symbolic value of the technology of any moral relevance?

ASCT is of great symbolic value. It represents the dissemination of phase II trial results, coverage without evidence, falsified data, high costs to the public and considerable, unnecessary harm to patients. Ethical issues are manifold [18,22-24,65,76,85,94,98] and many are already discussed in the aforementioned questions, e.g. Q1, Q2, Q13, Q14, Q16.

#### Questions "Moral Aspects of Methodological Choices" (Q24 to Q28)

##### Q24 Are there morally relevant issues related to the choice of end points in the assessment?

Endpoints in clinical trials or quality-of-life studies are highly affected by values. Depending on age, gender, socio-cultural context, family, religion, etc., valuing of survival, treatment-related adverse events and quality-of-life outcomes look very different. Long-term toxicities are more important to younger patients. Prolonged survival time or shorter treatment duration for equal survival or longer progression-free survival differ greatly in importance from one patient to another or from the viewpoint of physicians or researchers and patients. Survival probability is not only linked to statistical data, but also to ethical and psychological issues [10,44,99-101].

Hence, choosing and prioritizing endpoints also means choosing and prioritizing values. Survival-related dates are less important in palliative care than are quality-of-life issues, while quality of life is considered less important if there is a (marginal) chance of cure. In conclusion, individualizing and contextualizing evidence of endpoints is necessary [14,42,45,50,51,61,86,99,102-104]. But clinical trials and quality-of-life studies neglect the complexity of relationships by mostly considering one-dimensional measurement [38].

Choice of endpoints may also cause confounding of results. In ASCT, lack of experience is considered to be responsible for treatment-induced mortality and negative impact on long-term outcome [42,94,105].

Few publications address the choice of endpoints and their impact on economic evaluations even in the field of ASCT. However, long-term toxicities are highly relevant to cost-utility analyses [61,106].

##### Q25 Are there morally relevant issues related to the selection of meta-analysis and studies to be included in the HTA?

Seven papers highlight this topic [10,22,24,51,61,85,99]. Although the HTA report of Johnson et al. included the falsified Bezwoda results, the authors concluded that no definite recommendation could be drawn from the existing data [61]. Bergh criticizes the premature implementation of this technology based only on available data from phase I/II trials [51]. It is also stated that unethical clinical experiments and scientific misconduct cause long-lasting effects on trust in science [22,85]. Other authors argue that it is often very difficult to initiate a controlled trial if the intervention is believed to have a very high likelihood of being more effective than the control intervention [99].

##### Q26 Are the users of the technology in the studies representative of the users that will apply it in clinical practice?

The use of ASCT has changed widely since to the 1990s. According to the contemporary availability of treatment alternatives, the users of ASCT in the studies are not representative of the users in today's clinical practice.

Clinical practice could not reproduce the Bezwoda trial results because of falsified data. The results are far too exaggerated [22,85], and treatment-related mortality "was related to lack of experience with high-dose treatment". That is, low-volume centers observed lower survival than high-volume centers [100], making the trials only representative of highly specialized centers. Lee argued that the history of ASCT "might be relevant to other transplant programs and even other situations in medicine where patients accept substantial risks in the hope of a cure" [42].

Women who died from ASCT were not reasonably represented if only those patients that survived treatment were included in the trials (e.g. some quality-of-life studies) [43].

##### Q27 Are there morally relevant aspects with respect to the level of generalization?

To date, ASCT in breast cancer has been tested for both patient groups of relevance, i.e. high-risk breast cancer patients with multiple node positive disease and patients with distant metastases. However, particularly in the early phase II trials, it was likely that only selected patients were included in these studies [78]. Recruitment was frequently stopped in RCTs on metastatic breast cancer patients because of low willingness in patients to participate. Consequently, trials may be of poor statistical power [23].

##### Q28 Are there moral issues in research ethics that are important to HTAs?

Out of 18 papers discussing this issue [18,21,22,24,34,60,61,78,79,85,86,94,98,99,107-110] Smigel [78] gives the best summary of arguments: "More than 90% of women (1989-1993 treated with ASCT) did so outside of clinical trials (...). We are wasting valuable information by not getting those patients into clinical trials". The fact that the Bezwoda trials had not been approved by the local ethics and review committee together with the falsified publications led to mistrust, dramatically reducing enrolment of patients in prospective trials [108]. But the Bezwoda history did not change attitudes to planning, enforcement and reviewing of clinical studies - neither in the US nor in Europe [107]. No statement from an ethical point of view could be identified on the appropriateness of inclusion and exclusion criteria, control groups or control interventions.

#### Questions "Questions Related to Technology Assessment" (Q29 to Q33)

##### Q29 What are the reasons that this technology is selected to be assessed?

The dissatisfactory outcome of conventional chemotherapy in high-risk breast cancer is one reason for assessing ASCT [61]. Even today, metastatic breast cancer is considered to be an incurable disease. Since young women often die from this aggressive disease, physicians are pressurized to offer aggressive treatments [24]. Ten years after Bezwoda, effectiveness assessment still flags up some major ethical issues, but these have not been systematically assembled and synthesized. These issues, which are similar in other oncologic treatments, might be supplemented by some non-obvious ethical issues. Additionally, ASCT in breast cancer is, in fact, not media-covered. This was a primary criterion for the authors' work on methodological issues.

##### Q30 What are the interests of the persons participating in the technology assessment?

The persons involved in this review have been motivated by a desire to find (in the context of no financial or other conflict of interest) a conclusion for the affected women, in which the benefits and harms are balanced. Another objective is to find an eligible recommendation for financing or not financing treatment by health insurers. In addition, the involved persons are interested in finding and applying a feasible method of integrating ethical issues in effectiveness assessments.

##### Q31 At what time in the development of the technology is it assessed?

The assessment was done when the Bezwoda fraud was well-known, in clinical practice today only a few women are treated with ASCT and alternative treatments are available and used in clinical practice. This means the life cycle of the technology is already advanced.

Moreover, results of trials with more than 5-years' follow-up have been published, allowing the assessment of long-term overall and disease-free survival in breast cancer patients treated with ASCT. Nevertheless, the benefit of ASCT in special subgroups remains to be elucidated [1,111].

##### Q32 Are there related technologies that have or have not been assessed?

A major challenge is the assessment of cord blood transplantation [112-115]. While this technology is related to ASCT, cord blood cells are rarely used in clinical oncology. A few assessments on cord blood transplantations have been done already, but a comprehensive assessment of effectiveness and ethical issues with current evidence should be conducted. Additionally, the importance of all types of allogeneic stem cell transplantation [73] in breast cancer treatment has not been clarified to date. An assessment of such transplantations (in particular on the additional ethical issues and differences compared to autologous transplantation) could be helpful.

Modern imaging techniques in therapy monitoring (e. g. positron emission tomography (PET), PET/CT) are not yet standardized and still experimental in breast cancer treatment. In cases of misdiagnosis these technologies may cause wrong landmark changes in therapeutic management. Furthermore, they have not yet been assessed systematically from the ethical point of view - and these technologies are not related to ASCT.

##### Q33 What are the moral consequences of the HTA?

"Safety should be the first order of the day" should be the consequence of the assessment and impact on the quality of health care [116]. There are proposals for coverage of investigational treatments in terminal illness within clinical trials (rigorous protocol review process and thorough study overview comparing investigational treatment with standard therapy) if "life expectancy is limited, the investigational therapy provides the only possibility to preserve life, the treatment is prescribed by legitimate providers and performed in leading institutions and conventional therapy has failed [116]". Depending on individual cases, decisions on coverage are recommended.

Subsequent to the review of ethical issues, it is recommended that subgroup analysis is conducted from existing data. Analysis of individual patient data has recently begun [117,118]. It might be possible that, in the field of ASCT, although phase III trials and meta-analyses were undertaken, "expanded access ... would bring the doctors back into the drug development process and, rather than damage the clinical trial system, would greatly expand its effectiveness and value [35]".

Given that the ethical issues in the 1990s vary from those of today, a short summary of the currently relevant ethical issues of ASCT in breast cancer patients is presented in Table 5.

**Table 5:** Summary of currently relevant ethical issues in ASCT in breast cancer patients

Ethical issue
*Health technology*
Harm: Introduction of ASCT into clinical practice took place without further evaluation in clinical trials.
Harm: Incidence of severe side effects, risk of mortality and some negative impact on quality of life in the survivors
Trust: The only two trials which showed benefit in overall survival were found to be fraudulent (Bezwoda fraud). This caused significant effects on further research and the doctor-patient relationship as well as a lack of trust in oncology and the trial investigators. In consequence ASCT in breast cancer remains a stigmatized technology and of great symbolic value.
Uncertainty: It is unknown to date whether there is any evidence of a benefit of ASCT for some subgroups of breast cancer patients and, if so, whether this group would benefit more from an existing alternative.
External validity: The trials are only representative for highly specialised centres.
Alternatives: Safer procedures than ASCT do exist
*Patients*
Last chance therapy in metastatic breast cancer: As a consequence of this status patients postpone preparing for death. Attention is drawing away from more appropriate efforts to minimize symptoms and enhance the quality of life for terminally ill patients and their families. Recent publications discuss the right of patients to aggressive, toxic and expensive treatment - even untested with insufficient evidence on effectiveness and safety if it is potentially life-saving or life-prolonging.
Patient autonomy: Patients were faced with the decision to consent to a hopefully life prolonging but also potentially fatal treatment. There are some suggestions that patients are insufficiently informed and patient understanding is not always ensured.
Technological imperative: ASCT with the potential to benefit women with a life-threatening disease raises new hope for patients and physicians.

## Discussion

There are many ethical challenges related to the use of autologous stem cell transplantation in advanced breast cancer, both technological- and patient-related. Applying the normative framework formed by Hofmann's question list as axiological information [3,57,119] resulted in a comprehensive synthesis of qualitative arguments published in the literature. As the IQWiG is explicitly distinct from decision-making bodies thereby fulfilling the condition of the EUnetHTA HTA Core Model for medical and surgical interventions [2, page 84] which stated on cases that "the HTA organization is clearly separated from decision-makers it may be enough to describe the different values, attitudes and arguments that should be considered by the decision-makers". In this context, we prepared this review as a descriptive assessment. This may assist decision-makers in their decision process - in addition to the related results of the effectiveness assessment, and if applicable, on information related to the economic evaluation and the identified social, legal and organizational issues.

We did a descriptive assessment by adapting the methods of effectiveness assessment. While the adapted method on information retrieval is published [5] a more detailed presentation of the whole working process is scheduled to be a separate publication on methods of preparing systematic reviews on ethical issues as part of health technology assessments. Some of the arguments discussed in the publications are of historical value or are currently of less importance than they were in the 1990s, such as the premature implementation of the non-evaluated new technology in clinical practice.

Some results of past trials are fraudulent, but they continue to have an impact. For this reason, previously relevant ethical issues regarding this technology cannot be neglected.

The Socratic approach was chosen because of direct applicability by Hofmann's question list. In practice, and when a time restricted work flow is required, this has been an important advantage over to some other existing methodological approaches. Other approaches would also be able to address the central ethical dimensions, but some of the existing methodological approaches - with only one conceptual framework - would require additional content. Moreover, the Socratic approach has the advantage of being more comprehensive and paying attention to values related to the assessment itself.

All dimensions of Hofmann's question list could be addressed through the literature. No arguments were identified in the literature, which could not be assigned to one of the questions, and some issues identified could be assigned to more than one question. The findings of the ASCT example suggest that some questions could be summarized. In general, there is a tendency to lose first-order experiences of patients or patient groups by analyzing the (scientific) published literature. This is not the case in our example. Our retrieval results included some first-hand reports. Given that the assessed technology is not new, probably most reported first-hand experiences were covered by the scientific publications.

The synthesis was done in the context of European populations. From the viewpoint of other members of society, such as people with different social or cultural context, some issues may be missing from our presentation, or the presented issues may differ from their values. Our screening process excluded such variation in values. Furthermore, the presented arguments differently impact different healthcare systems and patient populations around the world.

## Conclusions

Regarding ASCT in breast cancer, some important ethical issues remain, which should be integrated into a comprehensive health technology assessment and be considered by both decision-makers and clinicians. The reflexive Socratic approach by means of the questions list by B. Hofmann proved to have good feasibility for synthesizing these issues. In our experience the 33 questions posed by Hofmann were very precise and exhaustive. Nevertheless, there could be other health technologies associated with ethical issues which are not being addressed or not being addressed in adequate detail by Hofmann's questions list.

The specific information retrieval identified the relevant literature with an appropriate sensitivity and precision. The selection process of identified publications on ethical issues that were included in the assessment and assigned to the questions and the ethical dimensions turned out to be a feasible approach.

In conclusion, our systematic literature review appears to be more qualified and comprehensive than statements from experts without a systematic review of relevant issues. This implies that conducting methodological high-quality reviews on ethical issues requires appropriate quality criteria and methodological standards to be defined and applied in ethics research and publication writing.

It may be useful to integrate the results of the systematic review on ethical issues into a HTA on the topic and to present them in a more reader-friendly format. Since our project was a pilot project an integration of the presented results in the HTA was not possible.

Further applications are recommended to validate this approach in a different context. There will be topics in which literature analyses are not justified or too few publications exist, and there are topics with inherent ethical conflicts, in which literature analyses are not appropriate. However, there are many topics and context situations in which descriptive reviews of ethical issues are appropriate.

## Authors' contributions

SD performed the information retrieval. All other work was jointly planned and elaborated by all authors. All authors read and approved the final manuscript.

## Pre-publication history

The pre-publication history for this paper can be accessed here:

http://www.biomedcentral.com/1472-6939/12/6/prepub

## References

[B1] Institute for Quality and Efficiency in Health Care Autologous stem cell transplantation for breast cancer 2009 Cologne: IQWiG 23101100

[B2] European Network for Health Technology Assessment Work package 4: HTA Core Model for Medical and Surgical Interventions. Version 1.0r 2008 Helsinki

[B3] Hofmann B Toward a procedure for integrating moral issues in health technology assessment Int J Technol Assess Health Care 2005 21 312 318 10.1017/S0266462305050415 16110710

[B4] Droste S Systematic search for information on ethical issues in HTA reports on medical technologies or interventions Z Evid Fortbild Qual Gesundhwesen 2008 102 329 343 http://portal.dimdi.de/de/hta/hta_berichte/hta062_summary_en.pdf (Update of the information retrieval chapter from: Droste S, Gerhardus A, Kollek R. [**Methods for the assessment of ethical aspects and moral concepts in society in short HTA reports an international survey**]. Niebüll: Medicombooks.de; 2003 10.1016/j.zefq.2008.03.001 19006921

[B5] Droste S Dintsios CM Gerber A Information on ethical issues in health technology assessment: how and where to find them Int J Technol Assess Health Care 2010 26 441 449 [additional material at http://www.journals.cambridge.org/thc2010029] 10.1017/S0266462310000954 20923585

[B6] Ackermann S Ethical problems of the use of stem cells in research and therapy Ethica 2001 9 227 243

[B7] Andrykowski MA Brady MJ Greiner CB Altmaier EM Burish TG Antin JH "Returning to normal" following bone marrow transplantation: outcomes, expectations and informed consent Bone Marrow Transplant 1995 15 573 581 7655384

[B8] Caocci G Pisu S Argiolu F Giardini C Locatelli F Vacca A Decision-making in adult thalassemia patients undergoing unrelated bone marrow transplantation: quality of life, communication and ethical issues Bone Marrow Transplant 2006 37 165 169 10.1038/sj.bmt.1705236 16299541

[B9] Domann U Effects of psycho-oncological interventions on survival and quality of life in patients undergoing stem cell transplantation] [Dissertation] 2005 Tübingen: University

[B10] Eddy DM Cost-effectiveness analysis: Is it up to the task? JAMA 1992 267 3342 3348 10.1001/jama.267.24.3342 1597918

[B11] Gottlieb S Bone marrow transplants do not help in breast cancer Br Med J 1999 318 1093 10.1136/bmj.318.7191.1093a PMC1115497 10213700

[B12] Hayes JD Clark EJ Breast cancer: clinical decision making Pharmacoeconomics 1993 4 226 228 10.2165/00019053-199304030-00007 10147131

[B13] Horton R After Bezwoda Lancet 2000 355 942 943 10.1016/S0140-6736(00)90006-0 10768426

[B14] Jones R Horowitz M Wall D Wingard JR Wolff S Documenting the case for stem cell transplantation: the role of evidence-based reviews and implications for future research. Statement of the Steering Committee for Evidence-based Reviews of the American Society for Blood and Marrow Transplantation (ASBMT) Biol Blood Marrow Transplant 2001 7 306 307 10.1016/S1083-8791(01)80002-1 11464974

[B15] Kletzel M Morgan E Frader J Ethical issues in stem cell transplantation J Hematother 1998 7 197 203 10.1089/scd.1.1998.7.197 9621253

[B16] Lesko LM Bone marrow transplantation: support of the patient and his/her family Support Care Cancer 1994 2 35 49 10.1007/BF00355238 8156256

[B17] Mahaney FX Bone marrow transplant for breast cancer: some insurers pay, some insurers don't J Natl Cancer Inst 1994 86 420 421 10.1093/jnci/86.6.420 8120914

[B18] Mello MM Brennan TA The controversy over high-dose chemotherapy with autologous bone marrow transplant for breast cancer: a cautionary tale about allowing politics and legal pressures to overwhelm science in evaluating new therapies Health Aff (Millwood) 2001 20 101 117 10.1377/hlthaff.20.5.101 11558695

[B19] Rettig R Jacobson P Farquhar CM Aubry WM False hope: bone marrow transplantation for breast cancer 2007 New York: Oxford University Press

[B20] Rubin BP Gratwohl A Reiter-Theil S Stem cell research: ethical potential of conflict and clinical level of expectation Onkologe 2003 9 128 139 10.1007/s00761-002-0459-9

[B21] Scott L New rules on therapy only fuel dispute Mod Healthc 1994 24 6 13 10133524

[B22] Sledge GW Jr Why big lies matter: lessons from the Bezwoda affair MedGenMed [Online Edition] 2000 2 10841629

[B23] Vogl DT Stadtmauer EA High-dose chemotherapy and autologous hematopoietic stem cell transplantation for metastatic breast cancer: a therapy whose time has passed Bone Marrow Transplant 2006 37 985 987 10.1038/sj.bmt.1705366 16708060

[B24] Welch HG Mogielnicki J Presumed benefit: lessons from the American experience with marrow transplantation for breast cancer Br Med J 2002 324 1088 1092 10.1136/bmj.324.7345.1088 PMC1123033 11991918

[B25] Belanger D Moore M Tannock I How American oncologists treat breast cancer: an assessment of the influence of clinical trials J Clin Oncol 1991 9 7 16 1985172 10.1200/JCO.1991.9.1.7

[B26] Farquhar C Marjoribanks J Basser R Hetrick S Lethaby A High dose chemotherapy and autologous bone marrow or stem cell transplantation versus conventional chemotherapy for women with metastatic breast cancer Cochrane Database Syst Rev 2005 CD003142 12535458 10.1002/14651858.CD003142

[B27] Farquhar C Marjoribanks J Basser R Lethaby A High dose chemotherapy and autologous bone marrow or stem cell transplantation versus conventional chemotherapy for women with early poor prognosis breast cancer Cochrane Database Syst Rev 2005 CD003139 16034886 10.1002/14651858.CD003139.pub2

[B28] Farquhar CM Marjoribanks J Lethaby A Basser R High dose chemotherapy for poor prognosis breast cancer: systematic review and meta-analysis Cancer Treat Rev 2007 33 325 337 10.1016/j.ctrv.2007.01.007 17382477

[B29] Andrykowski MA Psychosocial factors in bone marrow transplantation: a review and recommendations for research Bone Marrow Transplant 1994 13 357 375 8019460

[B30] Butow PN Dowsett S Hagerty R Tattersall MHN Communicating prognosis to patients with metastatic disease: what do they really want to know? Support Care Cancer 2002 10 161 168 10.1007/s005200100290 11862506

[B31] Cohen MZ Ley C Tarzian AJ Isolation in blood and marrow transplantation West J Nurs Res 2001 23 592 609 11569332 10.1177/019394590102300605

[B32] Comité Consultatif National d'Ethique pour les Sciences de la Vie et de la Santé Honnefelder L, Sturma D Commercialisation of human stem cells and other cell lines Jahrbuch für Wissenschaft und Ethik 12 2007 Berlin: de Gruyter 427 461

[B33] Conner-Spady BL Cumming C Nabholtz JM Jacobs P Stewart D A longitudinal prospective study of health-related quality of life in breast cancer patients following high-dose chemotherapy with autologous blood stem cell transplantation Bone Marrow Transplant 2005 36 251 259 10.1038/sj.bmt.1705032 15937502

[B34] Downs S Ethical issues in bone marrow transplantation Semin Oncol Nurs 1994 10 58 63 10.1016/S0749-2081(05)80045-2 8165379

[B35] Freireich EJ Gesme D Should terminally ill patients have the right to take drugs that pass phase I testing? Yes Br Med J 2007 335 478 479 10.1136/bmj.39244.451192.AD PMC1971171 17823187

[B36] Gahrton G Allebeck P, Jansson B Priorities for treatment with highly specialized technology: bone marrow transplantation Ethics in medicine: individual integrity versus demands of society 1990 New York: Raven Press 65 68

[B37] Giese W Adjudication of third party payment for high dose chemotherapy and bone marrow rescue in the treatment of breast cancer DePaul J Health Care Law 1996 1 205 242

[B38] Hjermstad MJ Kaasa S Quality of life in adult cancer patients treated with bone marrow transplantation: a review of the literature Eur J Cancer 1995 31a 163 173 10.1016/0959-8049(94)00464-G 7718320

[B39] Jacoby LH Maloy B Cirenza E Shelton W Goggins T Balint J The basis of informed consent for BMT patients Bone Marrow Transplant 1999 23 711 717 10.1038/sj.bmt.1701631 10218849

[B40] Kern D Kettner P Albrizio M An exploration of the variables involved when instituting a do-not-resuscitate order for patients undergoing bone marrow transplantation Oncol Nurs Forum 1992 19 635 640 1603678

[B41] Lambird PA College of American Pathologists Foundation Conference VII: ethical guidelines for biomedical technology development and application: the breast cancer paradigm. Introduction Arch Pathol Lab Med 1994 118 1074 1095 7979893

[B42] Lee SJ Fairclough D Antin JH Weeks JC Discrepancies between patient and physician estimates for the success of stem cell transplantation JAMA 2001 285 1034 1038 10.1001/jama.285.8.1034 11209174

[B43] McQuellon RP Craven B Russell GB Hoffman S Cruz JM Perry JJ Quality of life in breast cancer patients before and after autologous bone marrow transplantation Bone Marrow Transplant 1996 18 579 584 8879621

[B44] Nuscher Ford RE Kasprisin C, Snyder EL Psychosocial and ethical issues in bone marrow transplantation Bone marrow transplantation: a nursing perspective 1990 Arlington (VA): American Association of Blood Banks 129 152

[B45] Richter G Treatment of metastatic breast cancer: economic and ethical considerations Cancer Invest 1997 15 335 341 10.3109/07357909709039737 9246156

[B46] Singer DA Donnelly MB Messerschmidt GL Informed consent for bone marrow transplantation: identification of relevant information by referring physicians Bone Marrow Transplant 1990 6 431 437 2097013

[B47] Stiggelbout AM De Haes JCJM Patient preference for cancer therapy: an overview of measurement approaches J Clin Oncol 2001 19 220 230 11134216 10.1200/JCO.2001.19.1.220

[B48] Stiggelbout AM Jansen SJT Otten W Baas-Thijssen MCM Van Slooten H Van de Velde CJH How important is the opinion of significant others to cancer patients' adjuvant chemotherapy decision-making? Support Care Cancer 2007 15 319 325 10.1007/s00520-006-0149-z 17120070

[B49] Tarzian AJ Iwata PA Cohen MZ Autologous bone marrow transplantation: the patient's perspective of information needs Cancer Nurs 1999 22 103 110 10.1097/00002820-199904000-00001 10217025

[B50] Gratwohl A New developments in hematopoetic stem cell transplantation Ther Umsch 2002 59 571 576 10.1024/0040-5930.59.11.571 12498048

[B51] Bergh J Where next with stem-cell-supported high-dose therapy for breast cancer? Lancet 2000 355 944 945 10.1016/S0140-6736(00)90007-2 10768427

[B52] Fortanier C Mannoni-Chaine G Moatti J From bone marrow transplants to cell therapy: the uncertain trajectories of cooperation between medicine and industry Sci Soc Sante 1997 15 7 34

[B53] European Group on Ethics in Science and New Technologies Opinion on the ethical aspects of patenting inventions involving human stem cells 2002 Brussels: European Commission 12379991

[B54] Frick E Fegg MJ Tyroller M Fischer N Bumeder I Patients' health beliefs and coping prior to autologous peripheral stem cell transplantation Eur J Cancer Care (Engl) 2007 16 156 163 10.1111/j.1365-2354.2006.00725.x 17371425

[B55] Gahl K From stem cell research to stem cell therapy? Z Biopolit 2004 3 55 59

[B56] Chapman AR Frankel MS Garfinkel MS Stem cell research and applications: monitoring the frontiers of biomedical research 1999 Washington: American Association for the Advancement of Science and Institute for Civil Society

[B57] Hofmann B Is there a technological imperative in health care? Int J Technol Assess Health Care 2002 18 675 689 12391958

[B58] Edwards S Evaluating promising new treatments for life-threatening disease: implications of the HDC/ABMT experience for treating breast cancer Find Brief 2005 8 1 3 15672527

[B59] Kettner M Hauskeller C Is there a participative technology assessment on stem cell research in Germany? Human stem cells: therapeutic options, economic perspectives, media procurement 2002 Lengerich: Pabst Science 173 187

[B60] Mayer M When clinical trials are compromised: a perspective from a patient advocate PLoS Med 2005 2 1060 1063 10.1371/journal.pmed.0020358 PMC1255762 16220998

[B61] Johnson PWM Simnett SJ Sweetenham JW Morgan GJ Stewart LA Bone marrow and peripheral blood stem cell transplantation for malignancy Health Technol Assess 1998 2 1 154 9728295

[B62] Lanza R Rosenthal N The promise of stem cells: in the clouds of ethical controversies researchers struggle for the implementation of stem cell therapy. The scientific barriers are high, but not insuperable Spektrum Wiss 2004 34 41

[B63] Walshe R Schmitz N Diehl V Can corporatization contribute to quality assurance and cost control in the German hospital sector? A pilot project for stem cell transplantation Health Policy 1999 48 207 218 10.1016/S0168-8510(99)00051-2 11067039

[B64] Mazza P Prudenzano A Amurri B Palazzo G Pisapia G Stani L Myeloablative therapy and bone marrow transplantation in Jehovah's Witnesses with malignancies: single center experience Bone Marrow Transplant 2003 32 433 436 10.1038/sj.bmt.1704179 12900781

[B65] Jaggar SF Health insurance: coverage of autologous bone marrow transplantation for breast cancer Oncology (Williston Park) 1996 10 1329 8 1340 8882925

[B66] Gervais KG Priester R Mandates for unproven health care interventions Minn Med 1996 79 52 55 8637495

[B67] Statens Beredning för Utvärdering av Medicinsk Metodik SBU report on bone marrow transplantation: there are obvious ethical problems Lakartidningen 1991 88 3278 1943339

[B68] Mathews J NCI survey explores the M.D.'s perspective on ABMT trials J Natl Cancer Inst 1995 87 1510 1511 10.1093/jnci/87.20.1510 7563183

[B69] Davies S Bone marrow transplant raises issues of privacy Br Med J 1997 314 1356 PMC2126561 11644925

[B70] Hüsing B Engels EM Frietsch R Gaisser S Menrad K Rubin B Human stem cells 2003 Bern: TA-SWISS

[B71] Kelly D Ross S Gray B Smith P Death, dying and emotional labour: problematic dimensions of the bone marrow transplant nursing role? J Adv Nurs 2000 32 952 960 11095235

[B72] Rushing DA High-dose chemotherapy for breast cancer Ann Intern Med 1997 126 917 918 9163301 10.7326/0003-4819-126-11-199706010-00021

[B73] Jacobs P Hailey D MacLean N Allogeneic stem cell transplantation methods 1996 Edmonton: Alberta Heritage Foundation for Medical Research

[B74] Weitzner MA Lehninger F Sullivan D Fields KK Borderline personality disorder and bone marrow transplantation: ethical considerations and review Psychooncology 1999 8 46 54 10.1002/(SICI)1099-1611(199901/02)8:1<46::AID-PON332>3.0.CO;2-1 10202782

[B75] Healthcare America Plans Bossemeyer C Fiduciary duty, experimental medical treatment, standard of review, conflict of interest: sliding scale Benefits Q 2000 16 62 63 11183602

[B76] Brushwood DB Challenging denial of coverage for innovative therapy Am J Health Syst Pharm 1997 54 572 574 9066870 10.1093/ajhp/54.5.572

[B77] Holoweiko M When an insurer calls your treatment experimental Med Econ 1995 72 171 176 178-182 10151340

[B78] Smigel K Women flock to ABMT for breast cancer without final proof J Natl Cancer Inst 1995 87 952 955 10.1093/jnci/87.13.952 7629880

[B79] Stephenson J Researchers struggle with trials of stem-cell transplants for breast cancer JAMA 1997 277 1827 1829 10.1001/jama.277.23.1827 9185785

[B80] Jacobson PD Rettig RA Aubry WM Litigating the science of breast cancer treatment J Health Polit Policy Law 2007 32 785 818 10.1215/03616878-2007-030 17855717

[B81] Schulman KA Stadtmauer EA Reed SD Glick HA Goldstein LJ Pines JM Economic analysis of conventional-dose chemotherapy compared with high dose chemotherapy plus autologous hematopoietic stem-cell transplantation for metastatic breast cancer Bone Marrow Transplant 2003 31 205 210 10.1038/sj.bmt.1703795 12621482

[B82] Van Amerongen D Insurance payments for bone marrow transplantation in metastatic breast cancer N Engl J Med 2000 342 1138 1139 10.1056/NEJM200004133421515 10766588

[B83] Krause KJ Variations in insurance coverage for autologous bone marrow transplantation for breast cancer N Engl J Med 1994 331 329 332 10.1056/NEJM199408043310514 8068113

[B84] Daniels N Sabin JE Last chance therapies and managed care: pluralism, fair procedures, and legitimacy Hastings Cent Rep 1998 28 27 41 10.2307/3527569 9589291

[B85] Erikson J More fraud found in earlier Bezwoda data: should clinical trials on HDC/BMT for breast cancer continue? Oncol Times 2001 23 1 39-41

[B86] Leonard RCF The advancement of high-dose chemotherapy and dose intensification schedules Ann Oncol 1997 8 3 6 10.1023/A:1008217719071 9341958

[B87] Wynstra NA Breast cancer: selected legal issues Cancer 1994 74 1 Suppl 491 511 10.1002/cncr.2820741339 8004625

[B88] Molassiotis A Boughton BJ Burgoyne T van den Akker OBA Comparison of the overall quality of life in 50 long-term survivors of autologous and allogeneic bone marrow transplantation J Adv Nurs 1995 22 509 516 10.1046/j.1365-2648.1995.22030509.x 7499619

[B89] Andrykowski MA Cordova MJ Hann DM Jacobsen PB Fields KK Phillips G Patients' psychosocial concerns following stem cell transplantation Bone Marrow Transplant 1999 24 1121 1129 10.1038/sj.bmt.1702022 10578162

[B90] Peppercorn J Herndon J2 Kornblith AB Peters W Ahles T Vredenburgh J Quality of life among patients with stage II and III breast carcinoma randomized to receive high-dose chemotherapy with autologous bone marrow support or intermediate-dose chemotherapy: results from cancer and group B 9066 Cancer 2005 104 1580 1589 10.1002/cncr.21363 16118805

[B91] Byar KL Eilers JE Nuss SL Quality of life 5 or more years post-autologous hematopoietic stem cell transplant Cancer Nurs 2005 28 148 157 10.1097/00002820-200503000-00010 15815185

[B92] Mehnert A Scherwath A Schirmer L Schleimer B Petersen C Schulz-Kindermann F The association between neuropsychological impairment, self-perceived cognitive deficits, fatigue and health related quality of life in breast cancer survivors following standard adjuvant versus high-dose chemotherapy Patient Educ Couns 2007 66 108 118 10.1016/j.pec.2006.11.005 17320337

[B93] Van Luijn HEM Aaronson NK Keus RB Musschenga AW The evaluation of the risks and benefits of phase II cancer clinical trials by institutional review board (IRB) members: a case study J Med Ethics 2006 32 170 176 10.1136/jme.2002.001503 16507666 PMC2564477

[B94] Van Hoef M High-dose chemotherapy/bone marrow transplantation for breast cancer Oncol Times 2001 23 2 4

[B95] Peters WP Rogers MC Variation in approval by insurance companies of coverage for autologous bone marrow transplantation for breast cancer N Engl J Med 1994 330 473 477 10.1056/NEJM199402173300707 8289855

[B96] Rose LJ Autologous bone marrow transplants Health Aff (Millwood) 2002 21 308 10.1377/hlthaff.21.2.308 11900184

[B97] Sazama K Managing infectious or untested autologous blood components: the ethical dilemma of private rights versus public safety Arch Pathol Lab Med 2005 129 1212 1213 16196504 10.5858/2005-129-1212-MIOUAB

[B98] Fee K No place for politics: breast cancer treatment debacle shows what happens when politics interferes with science Mod Healthc 2000 30 48 50 11067549

[B99] Kamm M Changes of concise quality of life indices after high-dose chemotherapy 2004 Göttingen: University [Dissertation]

[B100] Vickberg SMJ DuHamel KN Smith MY Manne SL Winkel G Papadopoulos EB Global meaning and psychological adjustment among survivors of bone marrow transplant Psychooncology 2001 10 29 39 10.1002/1099-1611(200101/02)10:1<29::AID-PON482>3.0.CO;2-Y 11180575

[B101] Winer EP Quality-of-life research in patients with breast cancer Cancer 1994 74 1 Suppl 410 415 10.1002/cncr.2820741328 8004614

[B102] Curbow B Legro MW Baker F Wingard JR Loss and recovery themes of longterm survivors of bone marrow transplants J Psychosoc Oncol 1993 10 1 20 10.1300/J077V10N04_01

[B103] Langer S Abrams J Syrjala K Caregiver and patient marital satisfaction and affect following hematopoietic stem cell transplantation: a prospective, longitudinal investigation Psychooncology 2003 12 239 253 10.1002/pon.633 12673808

[B104] Lee SJ Joffe S Kim HT Socie G Gilman AL Wingard JR Physicians' attitudes about quality-of-life issues in hematopoietic stem cell transplantation Blood 2004 104 2194 2200 10.1182/blood-2003-07-2430 15198954

[B105] Sänger S Transplantations in oncology Onkologe 2001 7 1327 1328

[B106] Earle CC Chapman RH Baker CS Bell CM Stone PW Sandberg EA Systematic overview of cost-utility assessments in oncology J Clin Oncol 2000 18 3302 3317 10986064 10.1200/JCO.2000.18.18.3302

[B107] Armand JP The Bezwoda affair Bull Cancer 2000 87 363 364 10858047

[B108] Hagmann M Scientific misconduct: cancer researcher sacked for alleged fraud Science 2000 287 1901 1902 10.1126/science.287.5460.1901a 10755934

[B109] Rosoff PM Can underpowered clinical trials be justified? IRB 2004 26 16 19 10.2307/3563753 15281196

[B110] Weiss RB Rifkin RM Stewart FM Theriault RL Williams LA Herman AA High-dose chemotherapy for high-risk primary breast cancer: an on-site review of the Bezwoda study Lancet 2000 355 999 1003 10.1016/S0140-6736(00)90024-2 10768448

[B111] Nieto Y Nawaz S Shpall EJ Bearman SI Murphy J Jones RB Long-term analysis and prospective validation of a prognostic model for patients with high-risk primary breast cancer receiving high-dose chemotherapy Clin Cancer Res 2004 10 2609 2617 10.1158/1078-0432.CCR-03-0536 15102662

[B112] Blue Cross Blue Shield Association Transplanting adult patients with hematopoietic stem cells from placental and umbilical cord blood 2002 Chicago 11933941

[B113] Brinch L Husebekk A Funderud S Lyngstadaas A Use of hematopoietic stem cells from cord blood 2003 Oslo: The Norwegian Knowledge Centre for the Health Services (NOKC)

[B114] Swedish Council on Technology Assessment in Health Care Transplantation of stem cells from umbilical cord blood: early assessment briefs 2001 Stockholm

[B115] Yunkap Kwankam MM Hailey D Jacobs P Cord blood transplantation 1998 Edmonton: Alberta Heritage Foundation for Medical Research

[B116] Ader M Investigational treatments: coverage, controversy, and consensus Ann Health Law 1996 5 45 60 10164521

[B117] Berry DA Ueno NT Johnson MM Lei X Lopez V Caputo J High-dose chemotherapy with autologous stem-cell support versus standard-dose chemotherapy: Meta-analysis of individual patient data from 15 randomized adjuvant breast cancer trials Breast Cancer Res Treat 2007 106 Suppl 1 S5

[B118] Berry DA Ueno NT Johnson MM Lei X Smith DA Caputo J High-dose chemotherapy with autologous stem-cell support versus standard-dose chemotherapy: Meta-analysis of individual patient data from 6 randomized metastatic breast cancer trials Cancer Res 2008 69 2 Suppl S6113 10.1158/0008-5472.SABCS-6113

[B119] Grunwald A The normative basis of (health) technology assessment and the role of ethical expertise Poiesis Prax 2004 2 175 193

